# Variation in population levels of sedentary time in European adults according to cross-European studies: a systematic literature review within DEDIPAC

**DOI:** 10.1186/s12966-016-0397-3

**Published:** 2016-06-28

**Authors:** Anne Loyen, Maïté Verloigne, Linde Van Hecke, Ingrid Hendriksen, Jeroen Lakerveld, Jostein Steene-Johannessen, Annemarie Koster, Alan Donnelly, Ulf Ekelund, Benedicte Deforche, Ilse De Bourdeaudhuij, Johannes Brug, Hidde P. van der Ploeg, on behalf of the DEDIPAC consortium

**Affiliations:** Department of Epidemiology and Biostatistics, VU University Medical Center, EMGO+ Institute for Health and Care Research, De Boelelaan 1089a, 1081 HV Amsterdam, The Netherlands; Department of Movement and Sport Sciences, Faculty of Medicine and Health Sciences, Ghent University, Watersportlaan 2, 9000 Ghent, Belgium; Department of Public Health, Faculty of Medicine and Health Sciences, Ghent University, De Pintelaan 185, 9000 Ghent, Belgium; Physical activity, Nutrition and Health Research Unit, Department of Movement and Sport Sciences, Faculty of Physical Education and Physical Therapy, Vrije Universiteit Brussel, Pleinlaan 2, 1050 Brussels, Belgium; TNO Expertise Centre Lifestyle, Schipholweg 77-89, 2316 ZL Leiden, The Netherlands; Body@Work, EMGO+ Institute for Health and Care Research, VU University Medical Center, van der Boechorststraat 7, 1081 BT Amsterdam, The Netherlands; Department of Sports Medicine, Norwegian School of Sport Sciences, PO Box 4014, 0806 Ullevål Stadion Oslo, Norway; Department of Social Medicine, CAPHRI School for Public Health and Primary Care, Maastricht University, PO BOX 616, 6200MD Maastricht, The Netherlands; Centre for Physical Activity and Health Research, Department of Physical Education and Sport Sciences, University of Limerick, Limerick, Ireland; Department of Public and Occupational Health, VU University Medical Center, EMGO Institute for Health and Care Research, van der Boechorststraat 7, 1081 BT Amsterdam, The Netherlands; Sydney School of Public Health, The Charles Perkins Centre (D17), University of Sydney, 2006 Sydney, NSW Australia

**Keywords:** Adults, Assessment methods, Europe, Prevalence, Sedentary behaviour, Review

## Abstract

**Background:**

Sedentary behaviour is increasingly recognized as a public health risk that needs to be monitored at the population level. Across Europe, there is increasing interest in assessing population levels of sedentary time. This systematic literature review aims to provide an overview of all existing cross-European studies that measure sedentary time in adults, to describe the variation in population levels across these studies and to discuss the impact of assessment methods.

**Methods:**

Six literature databases (PubMed, EMBASE, CINAHL, PsycINFO, SportDiscus and OpenGrey) were searched, supplemented with backward- and forward tracking and searching authors’ and experts’ literature databases. Articles were included if they reported on observational studies measuring any form of sedentary time in the general population in two or more European countries. Each record was reviewed, extracted and assessed by two independent researchers, and disagreements were resolved by a third researcher. The review protocol of this review is registered in the PROSPERO database under registration number CRD42014010335.

**Results:**

Of the 9,756 unique articles that were identified in the search, twelve articles were eligible for inclusion in this review, reporting on six individual studies and three Eurobarometer surveys. These studies represented 2 to 29 countries, and 321 to 65,790 participants. Eleven studies focused on total sedentary time, while one studied screen time. The majority of studies used questionnaires to assess sedentary time, while two studies used accelerometers. Total sedentary time was reported most frequently and varied from 150 (median) to 620 (mean) minutes per day across studies and countries.

**Conclusions:**

One third of European countries were not included in any of the studies. Objective measures of European adults are currently limited, and most studies used single-item self-reported questions without assessing sedentary behaviour types or domains. Findings varied substantially between studies, meaning that population levels of sedentary time in European adults are currently unknown. In general, people living in northern Europe countries appear to report more sedentary time than southern Europeans. The findings of this review highlight the need for standardisation of the measurement methods and the added value of cross-European surveillance of sedentary behaviour.

**Electronic supplementary material:**

The online version of this article (doi:10.1186/s12966-016-0397-3) contains supplementary material, which is available to authorized users.

## Background

Sedentary behaviour has been defined as “any waking behaviour characterized by an energy expenditure of ≤ 1.5 metabolic equivalents (METs) while in a sitting or reclining position” [1] and is increasingly recognized as an important health risk. Time spent in sedentary behaviours is often referred to as sedentary time or its proxy sitting time. It is distinct from physical inactivity, which is defined as not meeting the physical activity recommendations [2]. Even though there is still debate about the health effects of sedentary time, a recent systematic review and meta-analysis reported a relationship between sedentary time and type 2 diabetes incidence, cancer incidence and mortality, cardiovascular disease incidence and mortality and all-cause mortality [3]. The risk of all-cause mortality has been reported to increase if adults sit more than approximately seven hours per day [4].

Recently, Hallal and colleagues estimated that 41.5 % of the adult world population spend more than four hours per day sitting [5]. Monitoring sedentary time at the population level is needed to study changes over time, identify and target populations at risk, and evaluate preventive strategies and policies. In addition, internationally comparable data will allow cross-country comparisons and benchmarking.

In 2013, twelve European Union Member States joined forces to establish the DEterminants of DIet and Physical ACtivity (DEDIPAC) Knowledge Hub. One of the aims of this Knowledge Hub is to enable a more standardized and continuous cross-European surveillance system to monitor dietary, physical activity and sedentary behaviours and its determinants over time and across the life course. This will help identify and target populations at risk in interventions and policies [6].

A first step towards a standardised surveillance system is to map the existing cross-European studies that monitor population levels of sedentary time. Moreover, the results of these studies could be used to estimate current sedentary time levels across European countries. In 2010, the World Health Organization (WHO) Regional Office for Europe made an overview of physical activity surveillance systems, also including sedentary time [7]. They concluded that even though there is increasing interest in assessing sedentary time across Europe, national surveys were not comparable due to differences in measurement methods, while international population surveillance efforts were scarce. Therefore, this study aims to provide an updated overview of solely cross-European studies, since these provide the opportunity for within-study country comparisons.

Four systematic reviews have been performed conjointly, focused on 1) physical activity in youth [8], 2) physical activity in adults [9], 3) sedentary time in youth [10], and 4) sedentary time in adults (the current review). The aim of the present review is to a) provide an overview of existing cross-European studies on sedentary time in adults (≥18 years), b) describe the variation in population levels of sedentary time according to these studies, and c) discuss the impact of study and measurement methods on these population levels.

## Methods

As described in the introduction, this systematic literature review is part of a set of four reviews. Because the four systematic reviews originate from the same project, have similar objectives (although for different behaviours and/or age groups) and share their methodology, the introduction-, methods- and discussion sections of the review articles have obvious similarities. The search, article selection, data extraction and quality assessment were conducted conjointly for all four reviews. Subsequently, the included articles were allocated to the appropriate review article (s). One article could be included in multiple reviews. If an article included both youth (<18 years) and adults (≥18 years) and presented stratified results, those stratified results were used in the appropriate review. If the article did not present stratified results, the article was allocated to the most appropriate review, based on the mean age (and age distribution) of the study sample. Before the search commenced, review protocols were written based on the “Centre for Reviews and Dissemination’s guidance for undertaking reviews in health care” [11], and registered in the PROSPERO database [12]. The review protocol of this review on sedentary time in adults is published under registration number CRD42014010335. The reporting of this systematic review adheres to the preferred reporting items of the PRISMA checklist (see Additional file 1).

### Search strategy

The search was conducted in June 2014 and updated on February29^th^, 2016. Six databases (PubMed, EMBASE, CINAHL, PsycINFO, SportDiscus and OpenGrey) were searched using similar search strategies, adapted to each database. The following search terms were used: ‘Physical activity’ OR ‘Sedentary behaviour’ AND ‘Europe’ (including all individual country names) AND ‘Countries’/’Multicountry’/’International’. Both the index terms and the title and abstract were searched and synonyms (e.g. for sedentary behaviour: sitting, screen time, etc.) were used. The complete search string can be found in Additional file 2. Based on the in- and exclusion criteria described below, search filters of the databases were used when possible, for example to select the appropriate publication period or language.

In addition, complementary search strategies were used. After the full-text review phase, the reference lists of the included articles were scanned (backward tracking) and a citation search was performed for the included articles (forward tracking) to identify potentially appropriate articles. Also, several experts in the field of physical activity and sedentary behaviour were contacted to provide additional articles. Finally, all authors involved in the four reviews were asked to search their own literature databases for appropriate articles. All additionally retrieved articles underwent the same selection process as the original articles - as described below.

### Article selection

All retrieved records were imported into Reference Manager 12 (Thomson Reuters, New York). Duplicates were hand-searched and removed. Records were included if they were journal articles, reports or doctoral dissertations (further referred to as ‘articles’) written in English. To be included, articles needed to report on observational studies conducted after 01-01-2000 (to avoid reporting outdated data) in the general, healthy population. In addition, articles were only included if they provided data for two or more European countries (as defined by the Council of Europe) [13].

With regard to sedentary time, articles were included if they reported total sedentary time (e.g. minutes/day), time spent sitting at school or work, time spent on screen time behaviours (e.g. TV viewing, using a computer), time spent sitting on motorised transport (e.g. in the car or public transport), and/or time spent at any other sedentary behaviour. Both subjective (e.g. questionnaires; usually assessing the time spent sitting) and objective (e.g. accelerometers; usually assessing the time spent at < 1.5 METs) measures were included.

Three researchers (AL, LVH, MV) were involved in the article selection, data extraction and quality assessment. For the title selection, the three researchers each independently reviewed 1/3 of the titles of the retrieved articles. For the abstract and the full-text selection, data extraction and quality assessment, the three researchers each covered 2/3 of the articles, so that each article was independently reviewed, extracted and assessed by two different researchers. Disagreement between the two researchers was resolved by the third researcher.

### Data extraction

A standardized data extraction file was used to extract data regarding the study characteristics, the study sample, the assessment methods, the reported outcomes, and the findings. We did not request the original data. The complete data extraction file can be found in Additional file 3.

### Quality assessment

A quality score was used to provide a general overview of the quality of the included articles. The ‘Standard quality assessment criteria for evaluating primary research papers from a variety of fields’ [14] was used for the assessment. The checklist consists of fourteen items to be scored ‘Yes’ (2 points), ‘Partial’ (1 point), ‘No’ (0 points) and ‘Not applicable’. The summary score was calculated as follows: Total sum ((number of ‘Yes’ x 2) + (number of ‘Partial’ x 1))/Total possible sum (28 – (number of ‘Not applicable’ x 2)). This instrument was chosen because it provides the opportunity to assess and compare the quality of different study designs, focuses on both the research and the reporting, and allows researchers to indicate that an item is not applicable, without affecting the total quality score. The complete quality assessment file can be found in Additional file 4.

## Results

**Fig. 1:**
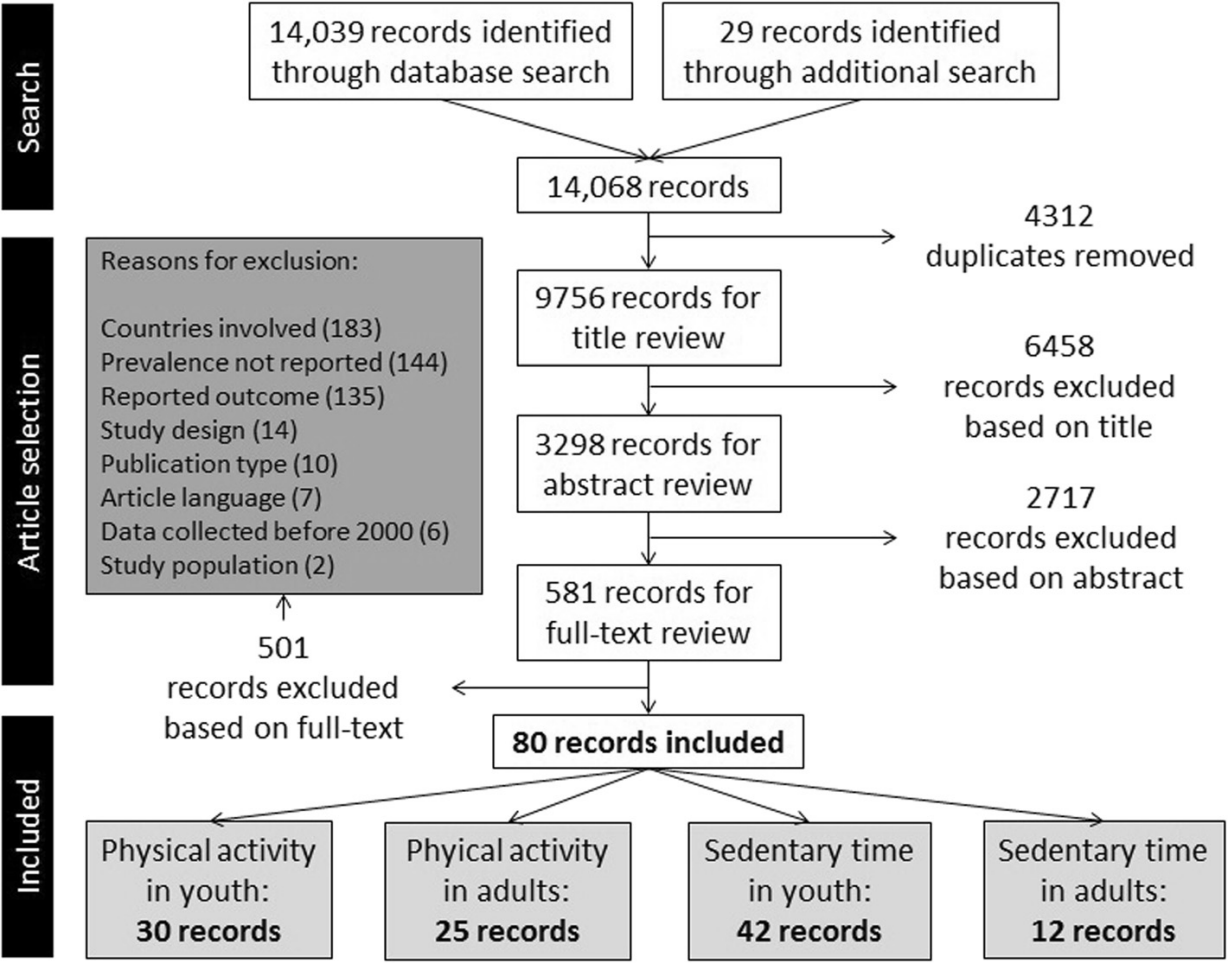
Flowchart of the combined review process

Figure 1 shows the flowchart of the combined review process for all four reviews. When combining the numbers of the original search and the update, 14,039 records were identified through the database search and 29 through the additional search, 9756 of which remained after duplicates were removed. After excluding 6458 records based on their title and 2717 based on their abstract, 581 records had their full-text reviewed. In this review phase, 501 records were excluded. Not including at least two European countries (*N* = 183), not reporting the prevalence numbers per country (*N* = 144) and not reporting a relevant outcome (*N* = 135) were the main reasons for exclusion. Finally, 80 records were identified as eligible to be included in the review article(s), of which twelve [15–26] were included in the current review on sedentary time in adults.

### Overview of the existing cross-European studies on sedentary time in adults

Table 1 shows the study and sample characteristics of the included articles. All articles were published between 2005 and 2016, with the majority published since 2013 [17–20, 23–26]. Six articles reported on the Eurobarometer surveys; one on the 2002 survey [15], two on the 2005 survey [16, 17], two on the 2013 survey [18, 19], and one on all three [20]. Except for one longitudinal study [25], all studies had a cross-sectional design. The quality scores ranged from 0.70 to 0.95 on a scale from 0 to 1. The studies included a minimum of 2 and a maximum of 29 countries, with the number of participants ranging from 321 to 65,790. While most studies included a broad age group, one study focused on students (mean age ranging from 22 to 38 years in the different samples) [23] and one study only included 21- and 25-year old young adults [25]. More than half of the articles reported using the IPAQ-short questionnaire to assess sedentary time [15–21], and minutes/day of total sitting time was the most reported outcome variable. None of the studies included Albania, Armenia, Azerbaijan, Bosnia and Herzegovina, Georgia, Iceland, Former Yugoslav Republic of Macedonia, Republic of Moldova, Montenegro, Russian Federation, Serbia, Switzerland, Ukraine and the microstates Andorra, Liechtenstein, Monaco and San Marino.

**Table 1:** Study information and sample characteristics of the articles included in the systematic review

Article	Study	Study design	Quality score (0–1)	Number of European countries	Number of European participants	Demographics				Sedentary time assessment method	Reported sedentary time outcome variables
						Age (range)	Gender (% female)	Level of education	BMI (mean)		
Eurobarometer (EB)											
Sjöström et al. (2006) [15]	EB 58.2 (2002)	CS	0.91	15	n.r.	15+	n.r.	n.r.	n.r.	Questionnaire; IPAQ-short	% sitting >6 h/day
Eurobaro-meter 64.3 (2006) [16]	EB 64.3 (2005)	CS	0.75	29	24,682	15+	52 %	25 % > 20 yrs old when stopped edu	n.r.	Questionnaire; IPAQ-short	Minutes/day sitting time
Bennie et al. (2013) [17]	EB 64.3 (2005)	CS	0.91	29	27,637	15-98	56 %	38 % >19 yrs edu	n.r.	Questionnaire; IPAQ-short	Minutes/day sitting time
Eurobaro-meter 80.2 (2014) [18]	EB 80.2 (2013)	CS	0.70	28	27,919	15+	n.r.	n.r.	n.r.	Questionnaire; IPAQ-short	% sitting ≤2 h30; 2 h31-5 h30; 5 h31-8 h30; >8 h31/day
Loyen et al. (2016) [19]	EB 80.2 (2013)	CS	0.95	28	26,617	18+	55 %	33 % 20+ years old when stopped edu	n.r.	Questionnaire; IPAQ-short	Minutes/day sitting time; % sitting >7.5 h/day
Milton et al. (2015) [20]	EB 58.2 (2002); 64.3 (2005) and 80.2 (2013)	CS	0.95	25	65,790	15+	52 %	18-19 mean years of edu	n.r.	Questionnaire; IPAQ-short	Minutes/day sitting time; % sitting >7.5 h/day
Other studies											
Bauman et al. 2011) [21]	IPS	CS	0.86	7	16,766	16-65	n.r.	n.r.	n.r.	Questionnaire; IPAQ-short	Minutes/day sitting time
Bourdeau-dhuij et al. (2005) [22]	/	CS	0.95	2	526	18+	65-66 %^a^	40-44 % higher edu^a^	23-26	Questionnaire; IPAQ-long	Minutes/week sitting time
Hainey et al. (2013) [23]	/	CS	0.82	2	887	23-38^a^ (mean)	30-62 %^a^	n.r.	n.r.	Questionnaire; Unspecified	Hours/week videogame playing
Lakerveld et al. (2015) [24]	SPOTLIGHT	CS	0.95	5	6,037		56 %	54 % higher edu	25	Questionnaire; Marshall	Minutes/day sitting time
Ortega et al. (2013) [25]	EYHS	LT	0.91	2	321	21 and 25	59-63 %^a^	n.r.	20-21^a^	Accelerometer; ActiGraph	Minutes/day sedentary time
Van Dyck et al. (2015) [26]	IPEN	CS	0.91	5	2,166	18-66	53 %	52 % college or higher	26	Accelerometer; ActiGraph (several models)	Minutes/day sedentary time

BMI = Body Mass Index; EB = Eurobarometer; IPS = International Prevalence Study; SPOTLIGHT = Sustainable Prevention of Obesity Through Integrated Strategies; EYHS = European Youth Heart Study; IPEN = International Physical activity and the Environment Network; CS = Cross-sectional; LT = Longitudinal; n.r. = not reported; yrs = years; edu = education; IPAQ = International Physical Activity Questionnaire; h = hours

^a^These publications only presented stratified demographics. The numbers shown here represent the range

### Variation in population levels of sedentary time in European adults

As discussed, several articles reported on the Eurobarometer surveys [15–20], including one article by Milton et al. that reported data on all three surveys [20]. To avoid presenting results from the same data twice, only this latter article will be used when describing reported levels of sedentary time. As Bulgaria, Croatia, Romania and Turkey were not included in the article by Milton et al., for these countries we will report data based on other articles reporting the same outcomes [17, 19].

The levels of sedentary time in adults across European countries can be viewed in Table 2, as a summary of the results reported in the included articles. To increase comparability across studies, we harmonised these results where this was possible. For example, one article reported sitting minutes per week instead of per day [22], we divided these numbers by seven to calculate minutes per day. Eight articles reported minutes/day of total sitting [17, 19–22, 24–26]. The lowest number (150 min per day (median)) was found in Portugal by Bauman et al. [21] using the IPAQ-short questionnaire, while the highest number (620 min/day(mean)) was obtained using the Marshall questionnaire by Lakerveld et al. in the United Kingdom [24]. The percentage of participants reporting sitting more than 7.5 h/day ranged from 9.7% in Spain (in 2013) to 42% in the Netherlands (in 2005) [20]. Dutch distance education students (mean age 38 years) reported the least hours/week playing videogames while Dutch regular education students (23 years old on average) and Scottish students (27 years old on average) were reasonably similar with 9.8 and 9.2 h/week spent playing videogames, respectively [23].

**Table 2:** Levels of sedentary time in adults across European countries. This table displays a summary of the results reported in the articles included in the systematic review

	Total sedentary time		Screen time
	Mean min/day total sedentary time [17, 19–22, 24–26] ^a^	% sitting > 7.5 h/day [19, 20]	Mean hours/week videogames playing [23]
Austria	2002: 306	2002: 23.7	
	2005: 303	2005: 18.1	
	2013: 329 [20]	2013: 19.7 [20]	
Belgium	2002: 321	2002: 25.6	
	2005: 343	2005: 28.6	
	2013: 301 [20]	2013: 19.6 [20]	
	300 (*M*) [21]		
	313 [22]		
	517 [24]		
	507 [26]		
Bulgaria	298 [17]	19.7 [19]	
	300 (*M*) [19]		
Croatia	308 [17]	22.7 [19]	
	300 (*M*) [19]		
Cyprus	2005: 368	2005: 35.3	
	2013: 298 [20]	2013: 19.9 [20]	
Czech Republic	2005: 386	2005: 37.7	
	2013: 327 [20]	2013: 26.8 [20]	
	360 (*M*) [21]		
	Site A: 486; Site B: 508 [26]		
Denmark	2002: 392	2002: 36.6	
	2005: 387	2005: 35.0	
	2013: 369 [20]	2013: 33.9 [20]	
	572 [26]		
Estonia	2005: 335	2005: 26.6	
	2013: 314 [20]	2013: 23.7 [20]	
	M: 455; F: 469 [25] *(25 yrs old)*		
Finland	2002: 362	2002: 33.2	
	2005: 346	2005: 31.9	
	2013: 339 [20]	2013: 25.0 [20]	
France	2002: 292	2002: 19.3	
	2005: 287	2005: 18.3	
	2013: 293 [20]	2013: 19.3 [20]	
	442 [24]		
Germany	East 2002: 340	East 2002: 28.0	
	2005: 314	2005: 22.9	
	2013: 284;	2013: 16.4;	
	West 2002: 337	West 2002: 26.5	
	2005: 337	2005: 25.1	
	2013: 298 [20]	2013: 19.9 [20]	
Greece	2002: 310	2002: 23.0	
	2005: 371	2005: 34.7	
	2013: 305 [20]	2013: 20.6 [20]	
Hungary	2005: 271	2005: 18.0	
	2013: 253 [20]	2013: 11.7 [20]	
	530 [24]		
Ireland	2002: 286	2002: 18.3	
	2005: 291	2005: 17.4	
	2013: 267 [20]	2013: 11.9 [20]	
Italy	2002: 335	2002: 25.9	
	2005: 268	2005: 14.4	
	2013: 267 [20]	2013: 11.3 [20]	
Latvia	2005: 273	2005: 20.6	
	2013: 290 [20]	2013: 18.3 [20]	
Lithuania	2005: 266	2005: 19.7	
	2013: 298 [20]	2013: 17.2 [20]	
	360 (*M*) [21]		
Luxembourg	2002: 333	2002: 27.5	
	2005: 324	2005: 26.5	
	2013: 322 [20]	2013: 26.0 [20]	
Malta	2005: 251	2005: 14.6	
	2013: 254 [20]	2013: 16.0 [20]	
Netherlands	2002: 357	2002: 28.4	9.8 (RE; mean age 23 yrs)
	2005: 410	2005: 42.0	3.9 (DE; mean age 38 yrs)
	2013: 376 [20]	2013: 34.4 [20]	
	569 [24]		
Norway	360 (*M*) [21]		
Poland	2005: 335	2005: 29.4	
	2013: 280 [20]	2013: 18.7 [20]	
Portugal	2002: 234	2002: 11.8	
	2005: 198	2005: 10.9	
	2013: 231 [20]	2013: 10.1 [20]	
	150 (*M*) [21]		
	330 [22]		
Romania	191 [17]	14.3 [19]	
	240 (*M*) [19]		
Slovak Republic	2005: 321	2005: 24.0	
	2013: 312 [20]	2013: 21.6 [20]	
Slovenia	2005: 309	2005: 26.6	
	2013: 249 [20]	2013: 14.7 [20]	
Spain	2002: 296	2002: 18.8,	
	2005: 281	2005: 16.5	
	2013: 265 [20]	2013: 9.7 [20]	
	300 (*M*) [21]		
	544 [26]		
Sweden	2002: 355	2002: 29.6	
	2005: 343	2005: 28.7	
	2013: 357 [20]	2013: 29.8 [20]	
	300 (*M*) [21]		
	M: 483; F: 469 [25] *(21 yrs old)*		
Turkey	305 [17]		
United Kingdom	GB 2002: 295	GB 2002: 19.2	9.2 (SC; mean age 27 yrs)
	2005: 325	2005: 23.3	
	2013: 300;	2013: 19.4;	
	NI 2002: 301	NI 2002: 23.0	
	2005: 299	2005: 19.5	
	2013: 279 [20]	2013: 18.5 [20]	
	620 [24]		
	499 [26]		

*M* = median; Site A = Olomouc; Site B = Hradec Kralove; M = males; F = females; yrs = years; RE = Regular Education; DE = Distant Education; GB = Great Britain; NI = Northern Ireland; SC = Scotland

a.Article [22] reported sitting minutes per week instead of per day. For comparison purposes, we divided these numbers by 7 to calculate minutes per day

**Fig. 2:**
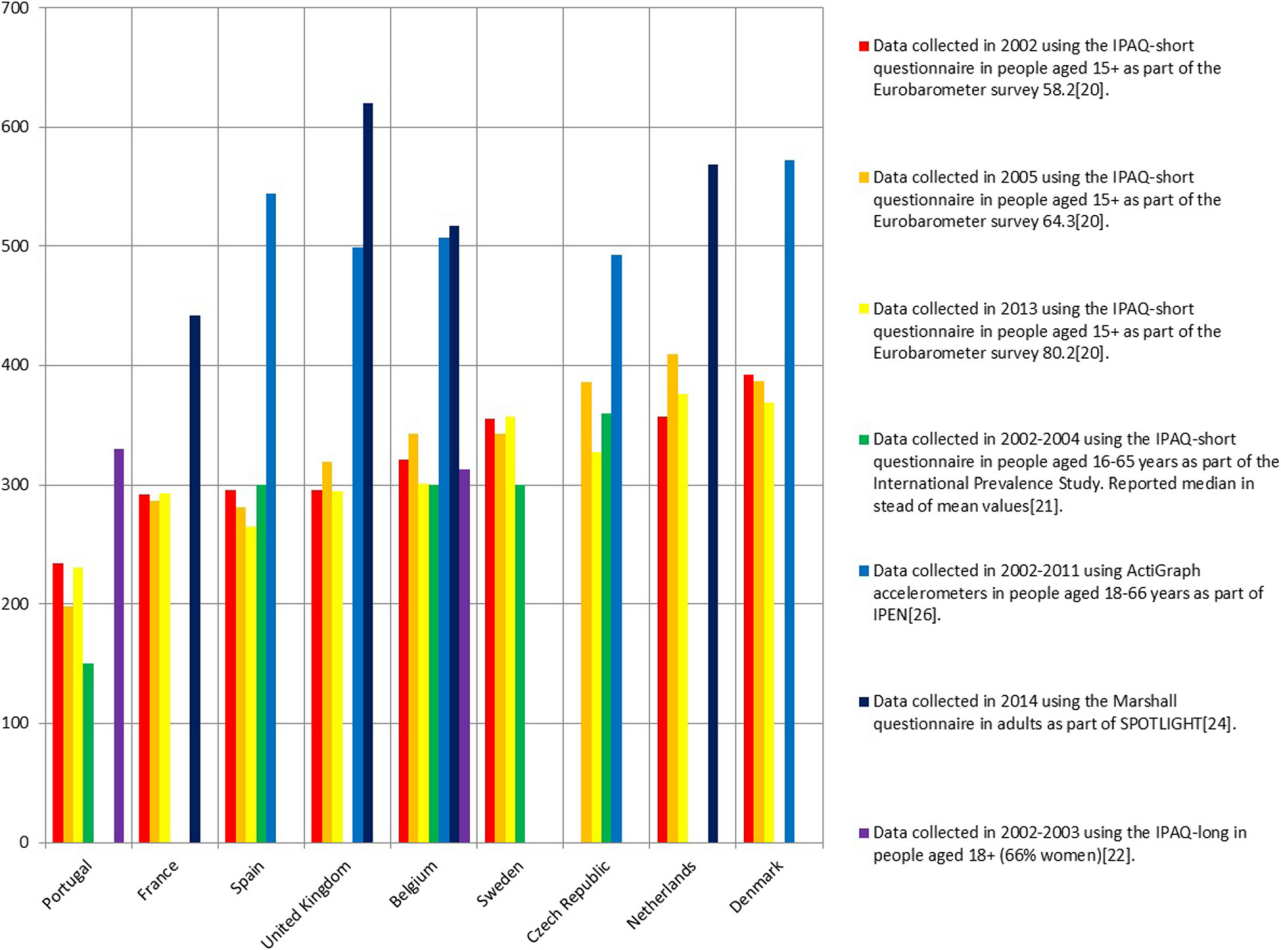
Minutes per day of total sitting time in adults across countries based on different articles. IPAQ = International Physical Activity Questionnaire; IPEN = International Physical activity and the Environment Network; SPOTLIGHT = Sustainable Prevention of Obesity Through Integrated Strategies

To provide a more accessible overview of the results, Fig. 2 shows the sitting minutes/day in nine different countries, based on seven studies, including the three Eurobarometer surveys. This outcome variable was the only outcome reported in multiple articles, and these countries and studies were included because they provided most data points. Overall, IPEN (using accelerometers) [26] and SPOTLIGHT (using the Marshall questionnaire) [24] report notably higher levels of sedentary time than the other studies. The results of the IPAQ-short studies [20, 21] are reasonably consistent in most countries. The results of the IPAQ-long questionnaire [22] seem comparable to the IPAQ-short questionnaire in Belgium, but not in Portugal. The United Kingdom shows the greatest within-country variation, with total sedentary time ranging from 295 up to 620 min/day.

### Variation in assessment methods and reported outcome variables

Table 3 provides an overview of the methods used to assess sedentary time and the outcomes reported in the included articles. In order to give a complete overview, all twelve included articles are considered again. Articles reporting on the same study are indicated in the table. Eleven articles reported on total sedentary time, while one article reported screen time (time spent videogame playing) [23]. Overall, five different assessment methods were used to report six different outcome variables. Two studies (reported in seven articles) used the IPAQ-short questionnaire [15–21], including the Eurobarometer surveys. Eight articles (based on five studies) reported minutes/day total sitting time [12, 13, 15, 18]. The articles based on the Eurobarometer surveys reported several different outcomes [15–20].

**Table 3:** Assessment methods and reported outcome variables in the articles included in the systematic review

	N	Reference (s)
**Study with multiple articles**		
EB 58.2	2	EB02 [15];[20]
EB 64.3	3	EB05 [16];[17];[20]
EB 80.2	3	EB13 [18];[19];[20]
**Assessment methods**		
*Questionnaire*	10	EB [15];[16];[17];[18];[19];[20], [21], [22], [23], [24]
IPAQ-short	7	EB [15];[16];[17];[18];[19];[20], [21]
IPAQ-long	1	[22]
Unspecified	1	[23]
Marshall	1	[24]
*Accelerometer (ActiGraph)*	2	[25], [26]
≤100 counts per minute	2	[25],[26]
**Reported outcomes**		
*Total sedentary time*	11	EB [15];[16];[17];[18];[19];[20], [21], [22], [24], [25], [26]
% sitting > 6 h/day	1	EB [15]
Minutes/day sitting time	8	EB [16];[17];[19];[20], [21], [24], [25], [26]
% sitting ≤ 2 h30;2 h31-5 h30;5 h31-8 h30;>8 h31/day	1	EB [18]
% sitting >7.5 h/day	2	EB [19];[20]
Minutes/week sitting time	1	[22]
*Screen time*	1	[23]
Hours/week videogame playing	1	[23]

EB = Eurobarometer 64.3; IPAQ = International Physical Activity Questionnaire; h = hours

## Discussion

The aims of this systematic literature review were to provide an overview of the existing cross-European studies on sedentary time in adults, to describe the variation in population levels of sedentary time, and to discuss the impact of assessment methods. We identified twelve articles that presented levels of sedentary time in European countries, reporting on six unique studies and three Eurobarometer surveys. The single-item IPAQ-short sitting question was the most frequently used assessment method, and minutes/day of total sitting time was reported most frequently as an outcome. Total reported sitting time varied from 2.5 h/day up to 10 h/day across studies and countries.

One third of the countries within the Council of Europe were not represented in any of the studies. Future cross-European studies should include these countries, in order to compare and benchmark them and to gain a complete picture of the population levels of sedentary time across Europe.

One article reported screen time, namely time spent playing videogames. Screen time, especially TV viewing time, is regularly used as a proxy for total sedentary time. However, research has shown that TV viewing time might not be representative for total sedentary time in adults [27, 28]. It is likely this also applies to time spent playing videogames. In addition, this study sample only included students in two European countries. Altogether, this study was not deemed suitable to report on population levels of total sedentary time in European adults.

Of the articles that reported on total sedentary time, two used an objective measure in the form of ActiGraph accelerometers. Even though these objective data have some limitations, including the high costs and the lack contextual information, they do provide a more valid and comparable estimation of the time spent sedentary. One of these studies included non-representative samples of five European countries as part of a larger international study, while the other study was conducted in small samples of young adults in two countries. This means that there is currently a limited amount of objective measurement of total sedentary time in adult samples across Europe. It should be noted, however, that accelerometer data is available in large scale national representative samples of adults in five European countries [29], but these were not included as they are single-country studies.

The remaining studies used questionnaires to assess self-reported sedentary time. Even though self-report questionnaires are regularly used in sedentary behaviour research, they have well-known limitations like recall- and social desirability bias, limiting their validity [30]. Moreover, participants from different cultures and/or countries might interpret questionnaire items differently. In addition, the questionnaires used in these studies only included one or two general questions about usual sitting time, without assessing the type of sedentary behaviour or the domain in which the sedentary behaviour took place, limiting their use in intervention development.

There is great variety in the assessment methods used and the outcomes reported in the included articles. Thus, it is difficult to draw conclusions about the population levels of sedentary time of European adults transcending the results of the separate articles. Sitting minutes/day is the only outcome variable that was reported in multiple studies and could thus be explored across studies and countries. The results of these studies seem reasonably consistent across those studies using the IPAQ-short questionnaire, while there are large differences across studies using other assessment methods. Specifically, the Marshall questionnaire and the ActiGraph accelerometer seem to record more sedentary time than the other assessment methods. Because of this, the population levels of sedentary time in Europe are currently unknown.

Because of the large differences between studies, cross-European comparison is only possible within studies. Of the identified studies, only the Eurobarometer surveys provide the opportunity to compare levels of sedentary time across a large number of countries. However, the Eurobarometer surveys have several disadvantages. First of all, they only include European Union Member States, and thus do not cover the whole of Europe. In addition, these surveys are not conducted for public health purposes, which is reflected in the irregularity by which questions about sedentary time are included. Sedentary time was assessed in 2002, 2005 and 2013, if and when it will be included again is not known. Finally, the Eurobarometer surveys use a single-item self-report question to assess sedentary time. Based on the Eurobarometer surveys, it seems that people living in northern European countries spend more time sitting down, while people in the south of Europe reported less sedentary time. In addition, based on a comparison of 2002, 2005, and 2013 data, Milton et al. concluded that levels of sedentary time might be decreasing, but these results should be viewed with caution since the response scale changed in the 2013 survey.

### Strengths and limitations

To our knowledge, this systematic literature review provides the first overview of all available cross-European studies reporting on the levels of sedentary time in European adults. The thorough and systematic review process is the main strength of this review. Before the search was conducted a review protocol was written and we adhered to this protocol throughout the process. Combining the search for the four reviews decreased the chance of missing articles, e.g. articles that were focussed on physical activity but also reported on sedentary time. The search was performed in six databases, including a grey literature-database, and multiple additional search strategies were used. Moreover, the article selection, data extraction and quality assessment were all conducted in duplicate, by independent researchers.

The search was performed in several databases and supplemented by several additional search strategies. However, the possibility remains that articles have been missed. Especially, articles could have been missed because we only included articles that were published in English. However, cross-European studies are likely published in the English language.

For these systematic literature reviews, we chose to only include studies that reported on at least two European countries, because in 2010, the WHO concluded that the results of national surveys were not comparable between countries. This means all studies with only national data were excluded even if they collected objective data, which might have been better comparable. It should be noted, however, that differences in data processing might have remained a problem for the comparability of the results from these studies. Pooling, harmonizing and comparing available objectively measured sedentary time data in national population based samples across Europe is a possible solution to this problem that is worth exploring in future studies.

We identified multiple articles that reported on Eurobarometer surveys. To avoid presenting results from the same survey twice, only the findings of the comparison article by Milton et al. is used to describe reported levels of sedentary time. Because the results reported in the different articles were only slightly different it is unlikely this will influence the conclusions of this review.

### Results of joint reviews

This review was part of a cluster of four reviews, focusing on the variation in population levels of 1) physical activity in youth [8], 2) physical activity in adults [9], 3) sedentary time in youth [10], and 4) sedentary time in adults (the current review). This review article included the smallest amount of articles, whereas the review on sedentary time in youth included more than three times as many articles. For both physical activity and sedentary time, more articles were focused on youth than on adults, indicating cross-European studies are more often conducted in youth than in adults. While the studies in adults as well as the youth studies on sedentary time predominantly used questionnaires as assessment method, the youth studies used accelerometers more frequently. All four reviews showed substantial variety in the assessment methods used and the outcome variables reported, making it difficult to compare across studies.

### Implications

The findings of these reviews highlight the need for standardisation of the measurement methods used to assess population levels of sedentary time in European adults. Ideally, objective measures such as accelerometers should be included as these provide more valid and comparable data, even though this might be challenging on such a large scale. Setting up a cross-European surveillance system with regular and state-of-the-art measures of physical activity and sedentary behaviour (and their determinants) in youth and adults could ensure the availability and continuity of high-quality data and involve countries that are currently excluded from cross-European studies. This could be achieved by integrating these measures into the Eurobarometer surveys, expanding and harmonising existing national studies, or by setting-up a new cross-European surveillance system. The results of such cross-European monitoring could inform public health campaigns and targeted interventions aiming to decrease sedentary time in Europe.

## Conclusion

There is currently no complete overview of the population levels of sedentary time in European adults. One third of European countries were not included in any of the studies. The number of cross-European studies with objective measures from adult samples is limited. Most identified studies used single-item self-report questions to assess sedentary time, without assessing the type of sedentary behaviour or the domain in which the sedentary behaviour took place. Within studies, there was large variation in the assessment methods, reported outcomes and, consequently, the findings, meaning that population levels of sedentary time in European adults are currently unknown. Generally, people in the north of Europe appear to report more time spent sitting than southern Europeans. The findings highlight the need for standardisation of the measurement methods and data processing used to assess sedentary time in Europe, and the development of a cross-European surveillance system with state-of-the-art measurement methods.

## Abbreviations

BMI, Body Mass Index; CS, Cross-sectional; DE, Distant Education; DEDIPAC, Determinants of Diet and Physical Activity; EB, Eurobarometer; Edu, Education; EYHS, European Youth Heart Study; F, Females; GB, reat Britain; H, Hours; IPAQ, International Physical Activity Questionnaire; IPEN, International Physical activity and the Environment Network; IPS, International Prevalence Study; LT, Longitudinal; M, Males; *M*, Median; MET, Metabolic equivalent; NI, Northern Ireland; n.r., not reported; RE, Regular Education; SC, Scotland; SPOTLIGHT, Sustainable Prevention of Obesity Through Integrated Strategies; WHO, World Health Organization; Yrs, Years

## Additional files

Additional file 1: The PRISMA checklist (PDF 212 kb) Additional file 2 The complete search string (PDF 175 kb) Additional file 3: Table S1. Data extraction file (XLSX 25 kb) Additional file 4: Table S2. The Quality assessment file (XLSX 12 kb)

## References

[1] Sedentary Behaviour Research Network. Letter to the editor: standardized use of the terms “sedentary” and “sedentary behaviours”. Appl Physiol Nutr Metab. 2012;37:540–2. 10.1139/h2012-024 22540258

[2] World Health Organization. Global Recommendations on Physical Activity for Health. World Health Organization. 2010. 26180873

[3] Biswas A, Oh PI, Faulkner GE, Bajaj RR, Silver MA, Mitchell MS (2015). Sedentary time and its association with risk for disease incidence, mortality, and hospitalization in adults: a systematic review and meta-analysis. Ann Intern Med.

[4] Chau JY, Grunseit AC, Chey T, Stamatakis E, Brown WJ, Matthews CE (2013). Daily sitting time and all-cause mortality: a meta-analysis. PLoS One.

[5] Hallal PC, Andersen LB, Bull FC, Guthold R, Haskell W, Ekelund U (2012). Global physical activity levels: surveillance progress, pitfalls, and prospects. Lancet.

[6] Lakerveld J, van der Ploeg HP, Kroeze W, Ahrens W, Allais O, Andersen LF (2014). Towards the integration and development of a cross-European research network and infrastructure: the DEterminants of DIet and Physical ACtivity (DEDIPAC) Knowledge Hub. Int J Behav Nutr Phys Act.

[7] WHO Regional Office for Europe. Review of physical activity surveillance data sources in European Union Member States. World Health Organization. 2010.

[8] Van Hecke L, Loyen A, Verloigne M, Van der Ploeg H, Lakerveld J, Brug J, et al. Variation in population levels of physical activity in European children and adolescents according to cross-European studies: a systematic literature review within DEDIPAC. Int J Behav Nutr Phys Act. Under revision. 10.1186/s12966-016-0396-4 PMC5399406 27350134

[9] Loyen A, Van Hecke L, Verloigne M, Hendriksen I, Lakerveld J, Steene-Johannessen J, et al. Variation in population levels of physical activity in European adults according to cross-European studies: a systematic literature review within DEDIPAC. Int J Behav Nutr Phys Act. Under revision. 10.1186/s12966-016-0398-2 PMC4924233 27350359

[10] Verloigne M, Loyen A, Van Hecke L, Lakerveld J, Hendriksen I, De Bourdeaudhuij I, et al. Variation in population levels of sedentary time in European children and adolescents according to cross-European studies: a systematic literature review within DEDIPAC. Int J Behav Nutr Phys Act. Under revision. 10.1186/s12966-016-0395-5 PMC4924322 27350043

[11] Centre for Reviews and Dissemination. Systematic reviews: CRD's guidance for undertaking reviews in health care. CRD, University of York, York. 2008.

[12] Centre for Reviews and Dissemination: PROSPERO database. http://www.crd.york.ac.uk/PROSPERO (2015). Accessed 2 May 2016.

[13] Council of Europe: our member states. http://www.coe.int/en/web/about-us/our-member-states (2015). Accessed 2 May 2016.

[14] Kmet LM, Lee RC, Cook LS. Standard quality assessment criteria for evaluating primary research papers from a variety of fields. Alberta Heritage Foundation for Medical Research. 2004.

[15] Sjostrom M, Oja P, Hagstromer M, Smith BJ, Bauman A (2006). Health-enhancing physical activity across European Union countries: the Eurobarometer study. J public Health.

[16] European Commission. Special Eurobarometer 246/Wave EB 64.3: Health and Food. European Commission. 2006.

[17] Bennie JA, Chau JY, van der Ploeg HP, Stamatakis E, Do A, Bauman A (2013). The prevalence and correlates of sitting in European adults - a comparison of 32 Eurobarometer-participating countries. Int J Behav Nutr Phys Act.

[18] European Commission. Special Eurobarometer 412/Wave EB 80.2: Sport and Physical Activity. European Commission. 2014.

[19] Loyen A, van der Ploeg HP, Bauman A, Brug J, Lakerveld J (2016). European Sitting Championship: Prevalence and Correlates of Self-Reported Sitting Time in the 28 European Union Member States. PLoS One.

[20] Milton K, Gale J, Stamatakis E, Bauman A (2015). Trends in prolonged sitting time among European adults: 27 country analysis. Prev Med.

[21] Bauman A, Ainsworth BE, Sallis JF, Hagstromer M, Craig CL, Bull FC (2011). The descriptive epidemiology of sitting. A 20-country comparison using the International Physical Activity Questionnaire (IPAQ). Am J Prev Med.

[22] Bourdeaudhuij ID, Teixeira PJ, Cardon G, Deforche B. Environmental and psychosocial correlates of physical activity in Portuguese and Belgian adults. Public Health Nutrition. 2007;8:886-95. 10.1079/phn2005735 16277805

[23] Hainey T, Westera W, Connolly TM, Boyle L, Baxter G, Beeby RB (2013). Students’ attitudes toward playing games and using games in education: Comparing Scotland and the Netherlands. Comput Educ.

[24] Lakerveld J, Ben Rebah M, Mackenbach JD, Charreire H, Compernolle S, Glonti K (2015). Obesity-related behaviours and BMI in five urban regions across Europe: sampling design and results from the SPOTLIGHT cross-sectional survey. BMJ Open.

[25] Ortega FB, Konstabel K, Pasquali E, Ruiz JR, Hurtig-Wennlof A, Maestu J (2013). Objectively measured physical activity and sedentary time during childhood, adolescence and young adulthood: a cohort study. PLoS One.

[26] Van Dyck D, Cerin E, De Bourdeaudhuij I, Hinckson E, Reis RS, Davey R (2015). International study of objectively measured physical activity and sedentary time with body mass index and obesity: IPEN adult study. Int J Obes (Lond).

[27] Healy GN, Clark BK, Winkler EA, Gardiner PA, Brown WJ, Matthews CE (2011). Measurement of adults’ sedentary time in population-based studies. Am J Prev Med.

[28] Sugiyama T, Healy GN, Dunstan DW, Salmon J, Owen N (2008). Is television viewing time a marker of a broader pattern of sedentary behavior?. Ann Behav Med.

[29] Wijndaele K, Westgate K, Stephens SK, Blair SN, Bull FC, Chastin SF (2015). Utilization and Harmonization of Adult Accelerometry Data: Review and Expert Consensus. Med Sci Sports Exerc.

[30] Atkin AJ, Gorely T, Clemes SA, Yates T, Edwardson C, Brage S (2012). Methods of Measurement in epidemiology: sedentary Behaviour. Int J Epidemiol.

